# Thermal imaging evaluation of paravertebral block for mastectomy in high risk patient: case report

**DOI:** 10.1007/s10877-014-9599-x

**Published:** 2014-07-25

**Authors:** Marcin Możański, Bartosz Rustecki, Bolesław Kalicki, Anna Jung

**Affiliations:** 1 Department of Anesthesiology and Intensive Care, Military Institute of Medicine, St. Szaserów 128, Warsaw, Poland; 2 Department of Pediatrics, Nephrology and Allergology, Military Institute of Medicine, St. Szaserów 128, Warsaw, Poland

**Keywords:** Paravertebral block, Mastectomy, Analgesia, Infrared photo, Monitoring

## Abstract

Thoracic paravertebral block is the technique of injecting local anesthetic adjacent to the thoracic vertebra close to where the spinal nerves emerge from the intervertebral foramina. It is effective in treating acute and chronic pain of unilateral origin from the chest and abdomen. This technique causes pain relief with pulmonary function preservation and great hemodynamic stability. 66 year old woman (156 cm, 80 kg, BMI 32) with chronic right heart failure, hypertension and obesity, on chronic oxygen therapy was presented for elective mastectomy due to breast cancer. She suffered from severe COPD and also bullous emphysema. FVC 1.59 l; FEV1 0.55 l; FEV1%FVC 34.6. The paravertebral block was performed using the multi-shot percutaneous technique with additional light general anesthesia. For confirmation, of proper analgesia range, control of temperature changes, using FLIR i7 infrared camera, was performed. Control photos were made 20 min after the blockade and then 10 min later. Infrared photo showed rise of temperature reading in every marked region. There were no hemodynamic and pulmonary complications postoperatively. Paravertebral block in combination with sedation creates excellent conditions for breast surgery procedures. Additional temperature changes monitoring performed with infrared camera may confirm proper range of analgesia needed to perform surgery. Great cardiovascular stability and very good pulmonary function preservation make this method excellent for high risk patients. Low complication rate is additional advantage. In our opinion this method is recommendable.

## Paravertebral block basics

Thoracic paravertebral block (TPVB) is a technique of injecting local anesthetic adjacent to the thoracic vertebra close to where the spinal nerves emerge from the intervertebral foramina. This results in ipsilateral somatic and sympathetic nerve blockade in multiple contiguous thoracic dermatomes above and below the site of injection [1, 2]. It is effective in treating acute and chronic pain of unilateral origin from the chest and abdomen [3–5].

TPVB is an old method of regional analgesia that was first described in the beginning of last century. In 1905 Hugo Sellheim from Leipzig described anatomical space lying alongside vertebral column. Following this discovery Sellheim and Lawen decided to inject small doses of local anesthetics into this space and to observe the effects [1].

This technique causes very good pain relief effect and also excellent pulmonary function preservation as well as great hemodynamic stability [6–8]. There is a short list of adverse effects and possible complications. Hypotension—4.6 %; Vascular puncture—3.8 %; Pleural puncture—1.1 % Pneumothorax—0.5 %. Coagulation disorders are rather relative contraindications for this technique [9].

At the Department of General, Oncological, Metabolical and Thoracosurgery first paravertebral block was performed in 2000 for analgesia of multiple rib fractures. Since then we performed a lot of PVBs mostly for breast surgery and thoracosurgery with very good analgesic effect and great preservation of pulmonary function.

## Case

66 year old woman (156 cm, 80 kg, BMI 32) presented for elective mastectomy due to breast cancer. She suffered from severe COPD and also bullous emphysema. FVC 1.59 l; FEV1 0.55 l; FEV1 %FVC 34.6. She was on home oxygen therapy. Chronic right heart failure, hypertension and obesity were the other problems.

The paravertebral block was performed using the multi-shot percutaneous technique at Th3, Th4, Th5 and Th6. We used 22 ml of 0.75 % ropivakaine with 0.2 mg of fentanyl. For confirmation, of proper analgesia range, control of temperature changes, using infrared camera was performed. Then light general anesthesia with laryngeal mask (LMA) and sevoflurane was performed.

**Fig. 1:**
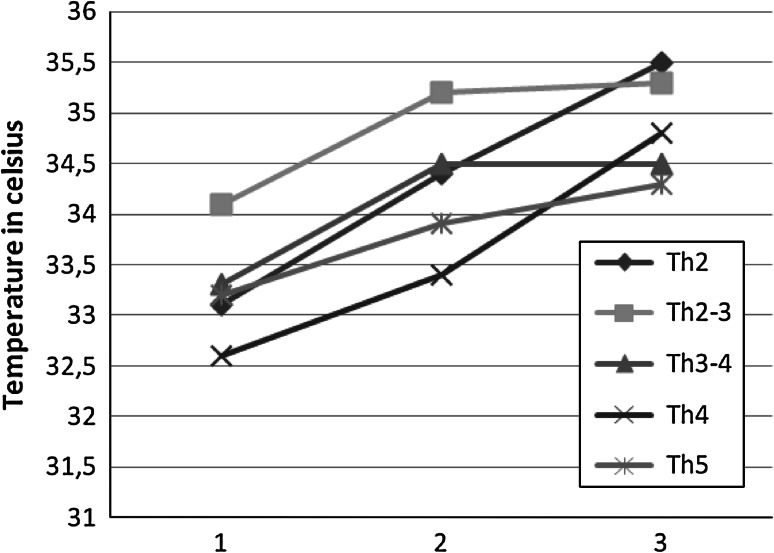
Temperature changes in check points. *1*—before blockade, *2*—20 min after blockade, *3*—30 min after blockade

Infrared photo were made from around 0.5 m distance with FLIR i7 camera, with preservation of proper standards [10]. Patient was uncovered while performing the exam. First picture was made before performing of paravertebral blockade procedure. Paravertebral multi-shot procedure took 6 min to perform. First control photo was made 20 min after the blockade and next 10 min after. Temperature average was marked for chosen representative area for every segment needed to be anesthetized to perform planed surgery. For every marked area we observed rise in temperature reading from infrared camera photo, which was indirect confirmation of proper paravertebral blockade. Data shown in Table 1, Fig. 1.

**Table 1:** Results from average infrared photo temperature reading

Marked segment	Time 0	Δ Time 0 to +20	Time + 20	Δ Time +20 to +30	Time + 30
seg Th2	33.1	1.3	34.4	1.1	35.5
seg Th2–3	34.1	1.1	35.2	0.1	35.3
seg Th3–4	33.3	1.2	34.5	0	34.5
seg Th4	32.6	0.8	33.4	1.4	34.8
seg Th5	33.2	0.7	33.9	0.4	34.3

Similar method of confirmation was previously used in S.P.S Cheema study of paravertebral analgesia. They have received confirmation of proper range of analgesia corresponding to ipsilateral temperature changes marked in infrared technique in six patients. Thus different level of paravertebral block was used in that study [11].

**Fig. 2:**
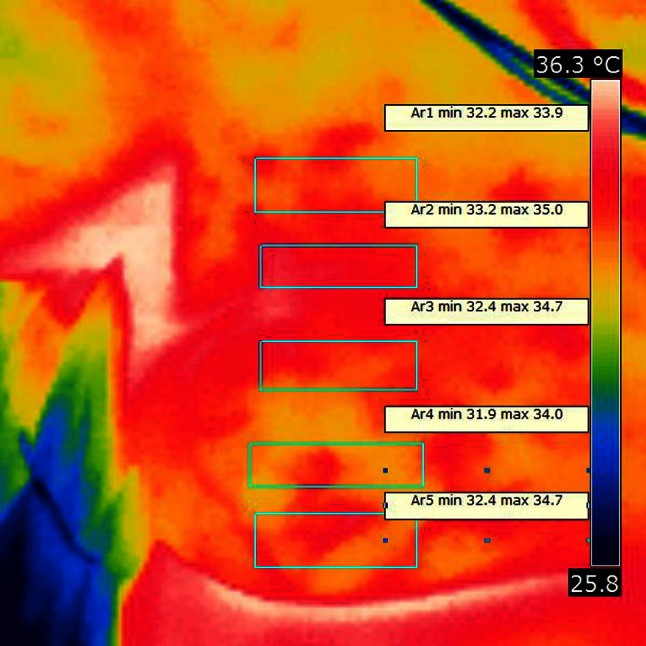
Infrared photo made before paravertebral blockade

**Fig. 3:**
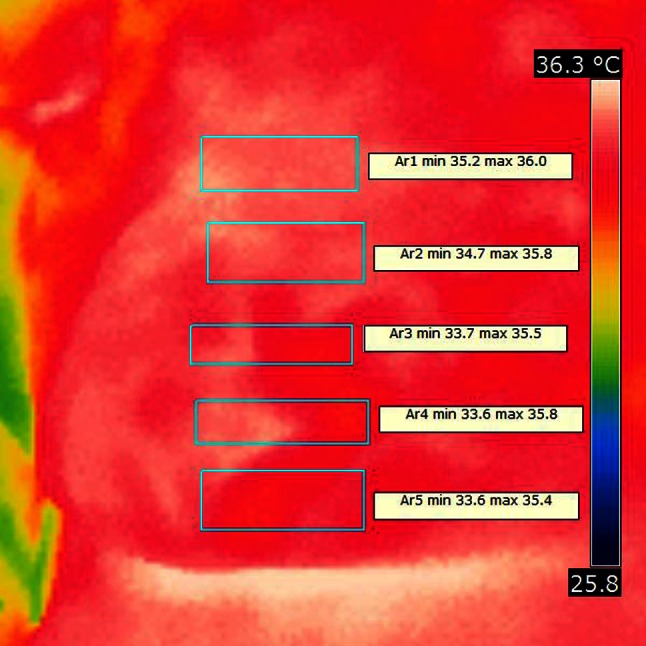
Infrared photo made 30 min after paravertebral blockade

Infrared photo showed rise of temperature count in every marked region after 20 min from injection. Latter infrared temperature reading also showed further increase of marked temperature confirming sympathetic nervous system blockade and indirectly proper range of analgesia. Example infrared pictures are in Figs. 2 and 3.

The patient received very good intraoperative analgesia with high cardiovascular stability and very good respiratory function preservation.

There were no hemodynamic and pulmonary complications postoperatively.

## Conclusion

Paravertebral block in combination with sedation creates excellent conditions for breast surgery procedures. Additional temperature changes monitoring performed with infrared camera may confirm proper range of analgesia needed to perform surgery. Great cardiovascular stability and very good pulmonary function preservation make this method excellent for high risk patients. Low complication rate is additional advantage. In our opinion this method is recommendable.
